# Decisional Regret Surrounding Dialysis Initiation: A Comparative Analysis

**DOI:** 10.1016/j.xkme.2023.100785

**Published:** 2023-12-20

**Authors:** Aditya S. Pawar, Bjorg Thorsteinsdottir, Sam Whitman, Katherine Pine, Alexander Lee, Nataly R. Espinoza Suarez, Paige Organick Lee, Anjali Thota, Elizabeth Lorenz, Annika Beck, Robert Albright, Molly Feely, Amy Williams, Emma Behnken, Kasey R. Boehmer

**Affiliations:** 1 Beth Israel Deaconess Medical Center, Boston, MA; 2 Community Internal Medicine, Mayo Clinic, Rochester, MN; 3 Knowledge and Evaluation Research (KER) Unit, Mayo Clinic, Rochester, MN; 4 College of Health Solutions, Arizona State University, Phoenix, AZ; 5 Human and Social Dimensions of Science and Technology, Arizona State University, Phoenix, AZ; 6 Health Services Research, Mayo Clinic, Rochester, MN; 7 VITAM Research Center on Sustainable Health, Québec Integrated University Health and Social Services Center (CIUSSS de la Capitale-Nationale), Québec, Canada; 8 Baylor College of Medicine, Houston, TX; 9 Nephrology and Hypertension, Mayo Clinic, Rochester, MN; 10 Bioethics, Mayo Clinic, Rochester, MN; 11 Palliative Care, Mayo Clinic, Rochester, MN

**Keywords:** Hemodialysis, peritoneal dialysis, shared decision making, patient-centered care

## Abstract

**Rationale & Objective:**

Dialysis comes with a substantial treatment burden, so patients must select care plans that align with their preferences. We aimed to deepen the understanding of decisional regret with dialysis choices.

**Study Design:**

This study had a mixed-methods explanatory sequential design.

**Setting & Participants:**

All patients from a single academic medical center prescribed maintenance in-center hemodialysis or presenting for home hemodialysis or peritoneal dialysis check-up during 3 weeks were approached for survey. A total of 78 patients agreed to participate. Patients with the highest (15 patients) and lowest decisional regret (20 patients) were invited to semistructured interviews.

**Predictors:**

Decisional regret scale and illness intrusiveness scale were used in this study.

**Analytical Approach:**

Quantitatively, we examined correlations between the decision regret scale and illness intrusiveness scale and sorted patients into the highest and lowest decision regret scale quartiles for further interviews; then, we compared patient characteristics between those that consented to interview in high and low decisional regret. Qualitatively, we used an adapted grounded theory approach to examine differences between interviewed patients with high and low decisional regret.

**Results:**

Of patients invited to participate in the interviews, 21 patients (8 high regret, 13 low regret) agreed. We observed that patients with high decisional regret displayed resignation toward dialysis, disruption of their sense of self and social roles, and self-blame, whereas patients with low decisional regret demonstrated positivity, integration of dialysis into their identity, and self-compassion.

**Limitations:**

Patients with the highest levels of decisional regret may have already withdrawn from dialysis. Patients could complete interviews in any location (eg, home, dialysis unit, and clinical office), which may have influenced patient disclosure.

**Conclusions:**

Although all patients experienced disruption after dialysis initiation, patients’ approach to adversity differs between patients experiencing high versus low regret. This study identifies emotional responses to dialysis that may be modifiable through patient-support interventions.

## Plain-Language Summary

As part of a quality improvement initiative in our dialysis practice, a patient stated, “I wish I never started dialysis.” This quote served as the catalyst for embarking on a research project with the aim to understand why patients living with end-stage kidney disease have regret about starting and continuing dialysis, a lifesaving but time-intensive measure. We surveyed and interviewed patients on the topic and learned that patients experiencing regret had a disrupted sense of self and blamed themselves for their need of dialysis. Patients with little to no regret demonstrated positivity and self-compassion. These findings will help health care professionals as they work with patients considering dialysis or having newly started dialysis.

Editorial, 100789

Current guidelines recommend that kidney replacement therapy option discussions should maximize shared decision-making (SDM);^1^, ^2^, ^3^ however, literature shows this is not consistently done.^4^, ^5^, ^6^ Ideally, the decision-making process to choose kidney replacement therapy options should fit patients’ values, beliefs, and preferences.^7^ Furthermore, for some patients, the choice of supportive care and forgoing dialysis may make the most sense, but this is frequently overlooked in the decision-making process.^8^

Decisional regret is an unpleasant emotion elicited when patients feel that an alternative choice may have resulted in a more favorable outcome than their current choice.^9^^,^^10^ The prevalence of decisional regret in patients with kidney failure varies between 7% and 61% in surveys.^11^, ^12^, ^13^, ^14^, ^15^, ^16^ Factors associated with decisional regret include lack of autonomy, with the decision-making process reflecting family and clinician preferences, and suboptimal preparation for and understanding of the dialysis process.^11^, ^12^, ^13^, ^14^, ^15^, ^16^ Decisional regret is less frequent among patients who discussed life expectancy, had a living will, and experienced greater decisional autonomy through SDM.^15^ Although studies reported potential factors contributing to decisional regret, there is a need for in-depth qualitative work in this space. Therefore, the aim of our study was to address the gap in understanding the differences in patient experiences and perspectives among those experiencing decisional regret to those with little to no decisional regret.

## Methods

This study was approved by the institutional review board at the Mayo Clinic (#18-000292), Rochester which is an integrated health care system with not-for-profit dialysis facilities. This was a sequential mixed-method study. The first phase was the application of a quantitative survey to inform purposeful sampling followed by a second phase of in-depth qualitative interviews as detailed in a previously published study.^17^

### Participant Recruitment

We approached 126 patients for participation, of whom 78 patients agreed to take part in a quantitative survey (Fig S1). Participants consented to participation in the follow-up interview if selected; however, patients were informed that they could decline the interview if they no longer wished to participate when recontacted. All patients were adults who consent to participation in English and were receiving ongoing maintenance dialysis (both in-center and home modalities) through 1 of 3 outpatient facilities. Patients with major barriers to consent such as cognitive impairment were excluded.

### Data Collection Procedures

#### Quantitative Survey

The paper-based survey was administered in-person by trained study personnel (Item S1). Patients could choose to return the survey immediately or via mail. The survey took patients 10-15 minutes to complete independently. Study staff were available for participant questions while they filled the survey and could read questions aloud to the participants if they preferred.

The survey was designed to assess patient characteristics including self-rated health, illness intrusiveness, and decisional regret about living life with dialysis. Illness intrusiveness asks participants to reflect on the extent to which their illness and/or treatment interferes with 13 aspects of their life.^18^ Decisional regret was measured using the decision regret scale (DRS),^19^^,^^20^ which asks participants to rate their agreement or disagreement on a 1-5 scale with 5 statements, as follows: “It was the right decision”; “I regret the choice that was made”; “I would go for the same choice if I had to do it over again”; “The choice did me a lot of harm”; and “The decision was a wise one.”

#### Quantitative Data Analysis

In DRS items 2 and 4 are reverse coded, such that for all items, a higher score indicates more regret. The scale is interpreted as a 0-100 range, in which 0 equals no regret and 100 would indicate the highest regret. To score the total scale, 1 is subtracted from each item’s value and then multiplied by 25. All items are then averaged for the total scale score. The DRS, commonly used in SDM studies, has only been used once with dialysis patients specifically.^14^ Illness intrusiveness scores are scored on a 1-7 scale; items marked not applicable are coded as 1. All items are totaled together for a composite score ranging from 13 to 91. This scale was developed in a dialysis population and has been widely used in chronic disease.^18^

We examined the correlation between patient-reported intrusiveness and DRSs because we planned to study in the follow-up interviews both aspects of illness intrusiveness and decisional regret. If these 2 measures were well correlated, a single study to examine differences between high or low regret and high or low illness intrusiveness would have been warranted. However, in the absence of such a correlation, 2 separate interview samples would be required. No power calculations were conducted before initiating the study. Ultimately, 2 studies were required with the illness intrusiveness data published elsewhere.^17^

Patients were sorted into quartiles based on their DRS scores. Patient demographics were summarized using percentages for categorical variables and quartile values for continuous variables. Differences between the lowest and highest quartiles for decisional regret who were interviewed were examined. No formal testing was done because of the low number of patients included in interviews. Statistical analysis was performed using SAS version 9.4.

#### Qualitative Interviews

Patients were purposively sampled for interviews to represent the lowest (DRS = 0) and highest (DRS of >25) quartiles of regret to identify predictors and modifiable contributors to decisional regret. The distribution of scores were skewed toward low to no regret. As such, we also chose to sample patients who answered, “I wish I had never started dialysis” to the question “How do you currently feel about starting dialysis?” regardless of their score on the DRS. With these patients included, 15 patients were categorized as high regret and are referred to as the high regret group (high regret). Patients who scored 0 on the DRS are referred to as the low regret group (low regret). For patients selected for in-depth interview, the duration of interviews was anywhere from 60 to 90 minutes.^21^ The interview guide is provided in Item S2. Interviews were voice recorded, transcribed verbatim, and documented with field notes. Interviews were conducted (NE, AB, SW, AT, and PO) in the dialysis center with option of interviewing in a private room or a patient’s home if they preferred. Interview training, progress, quality, and ensuring saturation was achieved were overseen by an experienced qualitative researcher (KRB). The interview team asked questions about patients’ experiences with decision-making, living with dialysis, and reasons for regret using a semistructured format (Item S2).^21^ No compensation was provided for filling the survey, but $25 was provided for participating in the interview. Interviewers had no previous relationship with patients interviewed and were in no way connected directly to their care.

#### Qualitative Data Analysis

There were a total of 21 interviews and transcripts analyzed using an adapted grounded theory approach^22^ facilitated with NVivo software. After reading all interview transcripts, 5 were coded line by line to create a codebook (SW, NE, AT, and PO). Codebook creation was supervised by KRB, BT, ASP, and KP interview transcripts were collectively coded, and disagreements were resolved after discussion. Information was summarized into themes by KRB, BT, and ASP across patients, and then examined to compare between the high regret and low regret. Codes and themes were discussed with coauthors to consider their perspectives and once agreement was reached, they were finalized to ensure rigor and credibility.

## Results

Out of 128 patients approached, 78 patients agreed to participate in survey. Overall, 35 patients were approached for interview, 20 were in lower quartile (low regret) and 15 were in high quartile (high regret). Twenty-one patients agreed to participate (8 high regret and 13 low regret), which was sufficient to achieve thematic saturation. Three patients in the low regret and no patients in the high regret were on home dialysis modality and rest were on in-center hemodialysis. Table 1 depicts patient demographics of the patients that were interviewed; full survey responses by quartile are provided in Table S1.

**Table 1:** Participant Characteristics and Survey Responses by DRS Quartiles

	Total (N = 21)	DRS Quartiles	
		Quartile 1: 0 (n = 13)	Quartile 4: >25 (n = 8)
**Age (y)**			
Median (IQR)	64.0 (36.0-70.0)	65.0 (52.0-70.0)	48.0 (32.5-68.5)
**Sex, n (%)**			
Female	11 (52.4%)	8 (61.5%)	3 (37.5%)
Male	10 (47.6%)	5 (38.5%)	5 (62.5%)
**Race, n (%)**			
White	14 (70.0%)	11 (84.6%)	3 (42.9%)
Black or African American	2 (10.0%)	0 (0.0%)	2 (28.6%)
Asian	1 (5.0%)	0 (0.0%)	1 (14.3%)
Other	3 (15.0%)	2 (15.4%)	1 (14.3%)
Missing	1	0	1
**Ethnicity, n (%)**			
Hispanic or Latino	2 (9.5%)	1 (7.7%)	1 (12.5%)
Not Hispanic or Latino	19 (90.5%)	12 (92.3%)	7 (87.5%)
**1. How would you describe your current health?, n (%)**			
Excellent	3 (14.3%)	1 (7.7%)	2 (25.0%)
Good	9 (42.9%)	5 (38.5%)	4 (50.0%)
Fair	4 (19.0%)	3 (23.1%)	1 (12.5%)
Very good	3 (14.3%)	2 (15.4%)	1 (12.5%)
Poor	2 (9.5%)	2 (15.4%)	0 (0.0%)
**2. How long have you received dialysis (y)?**			
Number of patients	20	13	7
Median (IQR)	2.0 (0.9-4.4)	3.5 (1.3-3.8)	1.0 (0.7-5.0)
**3. Was the decision to begin dialysis, n (%)**			
Planned	12 (60.0%)	8 (61.5%)	4 (57.1%)
Unplanned	8 (40.0%)	5 (38.5%)	3 (42.9%)
Missing	1	0	1
**4. Did you begin dialysis, n (%)**			
Inpatient or hospital setting	11 (52.4%)	6 (46.2%)	5 (62.5%)
Outpatient or clinic setting	10 (47.6%)	7 (53.8%)	3 (37.5%)
**5. Are you on a kidney transplant list?, n (%)**			
No	11 (55.0%)	8 (61.5%)	3 (42.9%)
Yes	9 (45.0%)	5 (38.5%)	4 (57.1%)
Missing	1	0	1
**6. Which of the following options were presented to you as treatment options to manage your kidney failure? (Check all that apply.) (choice** **=** **peritoneal dialysis), n (%)**			
Unchecked	9 (42.9%)	5 (38.5%)	4 (50.0%)
Checked	12 (57.1%)	8 (61.5%)	4 (50.0%)
**6. Which of the following options were presented to you as treatment options to manage your kidney failure? (Check all that apply.) (choice** **=** **home hemodialysis), n (%)**			
Unchecked	9 (42.9%)	4 (30.8%)	5 (62.5%)
Checked	12 (57.1%)	9 (69.2%)	3 (37.5%)
**6. Which of the following options were presented to you as treatment options to manage your kidney failure? (Check all that apply.) (choice** **=** **supportive care without dialysis), n (%)**			
Unchecked	18 (85.7%)	10 (76.9%)	8 (100.0%)
Checked	3 (14.3%)	3 (23.1%)	0 (0.0%)
**6. Which of the following options were presented to you as treatment options to manage your kidney failure? (Check all that apply.) (choice** **=** **kidney transplant), n (%)**			
Unchecked	12 (57.1%)	7 (53.8%)	5 (62.5%)
Checked	9 (42.9%)	6 (46.2%)	3 (37.5%)
**6. Which of the following options were presented to you as treatment options to manage your kidney failure? (Check all that apply.) (choice** **=** **none of the above), n (%)**			
Unchecked	18 (85.7%)	11 (84.6%)	7 (87.5%)
Checked	3 (14.3%)	2 (15.4%)	1 (12.5%)
**6. Which of the following options were presented to you as treatment options to manage your kidney failure? (Check all that apply.) (choice** **=** **choose not to answer), n (%)**			
Unchecked	21 (100.0%)	13 (100.0%)	8 (100.0%)
**7. Who mostly influenced your decision to start dialysis? (choice** **=** **primary care provider), n (%)**			
Unchecked	19 (90.5%)	12 (92.3%)	7 (87.5%)
Checked	2 (9.5%)	1 (7.7%)	1 (12.5%)
**7. Who mostly influenced your decision to start dialysis? (choice** **=** **nephrology provider), n (%)**			
Unchecked	6 (28.6%)	3 (23.1%)	3 (37.5%)
Checked	15 (71.4%)	10 (76.9%)	5 (62.5%)
**7. Who mostly influenced your decision to start dialysis? (choice** **=** **family), n (%)**			
Unchecked	19 (90.5%)	13 (100.0%)	6 (75.0%)
Checked	2 (9.5%)	0 (0.0%)	2 (25.0%)
**7. Who mostly influenced your decision to start dialysis? (choice** **=** **myself), n (%)**			
Unchecked	18 (85.7%)	12 (92.3%)	6 (75.0%)
Checked	3 (14.3%)	1 (7.7%)	2 (25.0%)
**7. Who mostly influenced your decision to start dialysis? (choice** **=** **other), n (%)**			
Unchecked	19 (90.5%)	12 (92.3%)	7 (87.5%)
Checked	2 (9.5%)	1 (7.7%)	1 (12.5%)
**7. Who mostly influenced your decision to start dialysis? (choice** **=** **choose not to answer), n (%)**			
Unchecked	21 (100.0%)	13 (100.0%)	8 (100.0%)
**7. (cont.) If other, please specify, n (%)**			
Death	1 (50.0%)	1 (100.0%)	0 (0.0%)
Friends	1 (50.0%)	0 (0.0%)	1 (100.0%)
Missing	19	12	7
**8. How do you currently feel about starting dialysis?, n (%)**			
Best decision I have made	4 (21.1%)	3 (23.1%)	1 (16.7%)
Not as bad as I thought it would be	5 (26.3%)	5 (38.5%)	0 (0.0%)
I thought it would be better, but I am okay with it	7 (36.8%)	5 (38.5%)	2 (33.3%)
I wish I had never started dialysis	1 (5.3%)	0 (0.0%)	1 (16.7%)
Other	2 (10.5%)	0 (0.0%)	2 (33.3%)
Missing	2	0	2
**8. (cont.) If other, please specify, n (%)**			
I’m alive.....	1 (50.0%)	0 (%)	1 (50.0%)
It’s okay, I will live with it	1 (50.0%)	0 (%)	1 (50.0%)
Missing	19	13	6
**9. I feel well prepared for what to expect with dialysis, n (%)**			
Strongly agree	7 (36.8%)	4 (30.8%)	3 (50.0%)
Agree	8 (42.1%)	7 (53.8%)	1 (16.7%)
Undecided	3 (15.8%)	1 (7.7%)	2 (33.3%)
Strongly disagree	1 (5.3%)	1 (7.7%)	0 (0.0%)
Missing	2	0	2
**10. How did your loved ones feel about your decision? n (%)**			
Highly satisfied	9 (47.4%)	8 (61.5%)	1 (16.7%)
Satisfied	8 (42.1%)	4 (30.8%)	4 (66.7%)
Uncertain	2 (10.5%)	1 (7.7%)	1 (16.7%)
Missing	2	0	2
**11. The cost of dialysis played a role in my decision to start dialysis, n (%)**			
Agree	4 (21.1%)	2 (15.4%)	2 (33.3%)
Undecided	2 (10.5%)	0 (0.0%)	2 (33.3%)
Disagree	3 (15.8%)	2 (15.4%)	1 (16.7%)
Strongly disagree	10 (52.6%)	9 (69.2%)	1 (16.7%)
Missing	2	0	2
**14. Has anyone on your care team specifically discussed your prognosis (life expectancy) with you?, n (%)**			
No	10 (52.6%)	5 (38.5%)	5 (83.3%)
Yes	9 (47.4%)	8 (61.5%)	1 (16.7%)
Missing	2	0	2
**15. How well informed do you feel about you current prognosis (life expectancy)?, n (%)**			
Very well informed	9 (45.0%)	8 (61.5%)	1 (14.3%)
Somewhat informed	7 (35.0%)	3 (23.1%)	4 (57.1%)
Unsure	3 (15.0%)	2 (15.4%)	1 (14.3%)
Somewhat uninformed	1 (5.0%)	0 (0.0%)	1 (14.3%)
Missing	1	0	1
**16. How do you expect your health to be in 12 months from now?, n (%)**			
Much better	6 (28.6%)	4 (30.8%)	2 (25.0%)
Somewhat better	5 (23.8%)	2 (15.4%)	3 (37.5%)
The same	8 (38.1%)	5 (38.5%)	3 (37.5%)
Worse	2 (9.5%)	2 (15.4%)	0 (0.0%)
**17. If you were seriously ill, would you prefer care to, n (%)**			
Extend life, even if it meant more pain and discomfort	12 (63.2%)	8 (61.5%)	4 (66.7%)
Relieve pain and discomfort, even if it meant not living as long	7 (36.8%)	5 (38.5%)	2 (33.3%)
Missing	2	0	2
**20. It was the right decision, n (%)**			
Strongly agree	14 (66.7%)	13 (100.0%)	1 (12.5%)
Agree	3 (14.3%)	0 (0.0%)	3 (37.5%)
Neither agree nor disagree	4 (19.0%)	0 (0.0%)	4 (50.0%)
**21. I regret the choice that was made, n (%)**			
Agree	1 (4.8%)	0 (0.0%)	1 (12.5%)
Neither agree nor disagree	6 (28.6%)	0 (0.0%)	6 (75.0%)
Disagree	1 (4.8%)	0 (0.0%)	1 (12.5%)
Strongly disagree	13 (61.9%)	13 (100.0%)	0 (0.0%)
**22. I would go for the same choice if I had to do it over again, n (%)**			
Strongly agree	14 (66.7%)	13 (100.0%)	1 (12.5%)
Agree	2 (9.5%)	0 (0.0%)	2 (25.0%)
Neither agree nor disagree	5 (23.8%)	0 (0.0%)	5 (62.5%)
**23. The choice did me a lot of harm, n (%)**			
Strongly agree	2 (9.5%)	0 (0.0%)	2 (25.0%)
Neither agree nor disagree	3 (14.3%)	0 (0.0%)	3 (37.5%)
Disagree	3 (14.3%)	0 (0.0%)	3 (37.5%)
Strongly disagree	13 (61.9%)	13 (100.0%)	0 (0.0%)
**24. The decision was a wise one, n (%)**			
Strongly agree	13 (61.9%)	13 (100.0%)	0 (0.0%)
Agree	4 (19.0%)	0 (0.0%)	4 (50.0%)
Neither agree nor disagree	4 (19.0%)	0 (0.0%)	4 (50.0%)

Abbreviations: cont., continued; DRS, decision regret scale; IQR, interquartile range.

### Qualitative Findings

Four themes emerged from the comparative analysis: mindset, reconciling life while receiving dialysis, biographic disruption, and understanding and learning of treatment requirements (Patients’ quotes, Table 2). There were themes that were expressed by both groups, such as inability to travel and dietary restrictions.

**Table 2:** Themes and Illustrative Patient Quotations

Themes	Illustrative Quotations	
	Low Regret	High Regret
**Mindset**	*“Well, like I said, it was—my world was shattered into a million pieces, but it was not lacking in a positive approach. I was mainly just trying to keep myself alive. At worst, I would just be alive. At best, maybe, something great might happen, so I could just keep pushing forward. Don't give up hope.” (30s, in-center HD)* *“I was the youngest there and it doesn’t matter what age you are, don’t stop making plans for yourself and making goals. Continue on with your life and be positive about it.” (60s, in-center HD)* *“I don’t know what he thought it was, but I said, “You’re just layin’ in a chair for 3 hours.” That’s about it. There’s nothing painful about it or anything like that. I think they just don’t know what they’re getting into. No idea, but other than that.. It’s not so bad. It’s just the time that you spend doin’ it.” (70s, in-center HD)*	*“I had to quit work. I knew a lotta my time was going to be consumed with dialysis, but I’d rather have that than just not—be worse.” (20s, in-center HD)* *“You can go through until you tired. That’s one chance. Keep waiting, waiting. While we’re waiting, we have dialysis. That’s how lucky we are. It’s going through the hard parts. You have to do it. That’s what I learn.” (Living with dialysis and waiting for death or transplant) (60s, in-center HD)* *“That’s what they recommend so we have to follow the recommendation of the doctor. They want us do the dialysis, that’s what I have to do that in order to survive. That’s what it is.” (60s, in-center HD)*
**Reconciling life while receiving dialysis**	*“Well, the diet is a real shock. It’s very hard, although I’ve learned that now and then you can go off the diet and not be too—at first I was real strict about it, and I lost 10 pounds practically. Now I’ve got back that weight.” (70s, in-center HD)* *“It’s not dialysis, per se. The dialysis is what keeps you alive. You start feeling better, and so you can function more as a normal person, which I try to do all the time. Whenever there's any little glitches, I always try to find solutions. How can I make this, so I can be more like a normal person.” (60s, in-center HD)* *“Continue making goals for yourself and keep achieving things cuz it’s not the end, it’s not the end of your life. It doesn’t, that chair ties you there 3 days a week but you can still do things.” (Male in 60s, in-center HD)*	*“I feel like a—I don’t know but I feel like this a last resort though. I feel like I have to obligate to do that in order to survive. That’s how I feel. This is I can live not without that. I learn that. This is the obligation.” (Male, 60s, in-center HD)* *“My kids. Their smile or just seeing them. Waking up to them every day. If I’m having a bad day or upset for whatever reason, they just—By keeping me alive, because I’m pretty sure if I wouldn’t have chosen this, I’m sure I wouldn’t be here right now.” (20s, in-center HD)*
**Biographical disruption**	*“Yeah. You have to know a month ahead of times to let the social worker here know, so she can set up dialysis appointments for you wherever you’re going. You almost have to fly.” (Female, 70s, in-center HD)* *“I still have my days that I don’t feel good at all, but I have way more good days than bad days and I like that I can continue to work full time. It’s important to me. Work is a good distraction for me from life and all that it brings. I enjoy being at work and I enjoy what I do. It’s important to me.” (40s, in-center HD)* *“Well, it doesn't take much, but the real joy I guess is I have this nice little family, a husband, and a dog that I love dearly, both of them. I can spend time with them, and do things. If we want to go somewhere, or go out to eat, or do whatever, and that there’s somebody there to go do these things with that I’m not so isolated, so alone that there’s not somebody to go, I want to go to this place on the weekend or that place.” (60s, in-center HD)*	*“it’s probably not so much to my family. It’s probably more me, psychological, that I feel like a burden, because I can’t go on trips or if I wanna go on trips, just takes me a long time to get everything set up. I don’t go on the boat as much anymore, cuz I don’t wanna say I’m scared, but things can happen. If I fell out of the boat into that deep water, what’s gonna happen? Especially if this thing gets underneath the water and all that water comes up in there?” (30s, in-center HD)* *“What I have planned for the future it’s not a way of life anymore, it’s an obstacle that needs to be removed. With what I’ve got in mind? Yeah, this is in my way. I eventually want to open my own business. It’s kinda hard to do that when you’re here 3 days a week. My girlfriend and I are talking about getting married. Sorry honey we can’t go that on our honeymoon. I got to go to stupid dialysis. That’s not fair.” (30s, in-center HD)* *“I used to be—my last job was working in ‘Town X’ out in in ‘Company Y.’ I was gonna be a supervisor there, but (crosstalk 03:42)—yes. Then I got sick. I got worse. I started feeling really bad, and I just—I couldn’t stay at my job.”(20s, in-center HD)* *“It’s a lot of change. Life change. Because you get stuck with this one. You cannot do anything more like we normally did in the past.” (60s, in-center HD)* *“Just I miss my kids. It’s hard to be away from them, especially I have a XY-month-old(infant). It’s really hard being away from her in the mornings.” (20s, in-center HD)*
**Understanding and learning treatment requirements**	*“I didn’t understand at first, cuz they would ask me. I didn’t know. Then slowly I understood what they were askin’ because if you pulled too much off. it cramps. You get dizzy. You get to know your own limits. It’s important to pay attention to what they’re doin’, and the rate of flow, because that can affect a lot about how I feel. I’m the only person doin’ that right now here that they are taking care of right now so it’s just not really, but I had talked to other people online and so I knew that it was a possibility. I don’t really know that there was—I think that it’s a lot of work that I was, I knew it would be work but it’s definitely a full time gig.” (50s, in-center HD)*	*“I feel like it was all my—I feel like at that point, it was my fault, because I should’ve been more prepared to that point. I should’ve paid more attention to what was really going on. Like I said, I felt invincible.” This ain’t nothing.” (Male, 30s, in-center HD)* *I don’t think education—you could go too far with education, especially like this. Experiencing what I’m going through. I’m telling you. They can use that for letting somebody else know. At first nothing, cuz I had no idea what—they explained to me, but without seeing the process, I had no idea. Then once they put this thing in(access), and I start doing it, it got real.” (30s, in-center HD)* *I feel a lot better now just because learning all of this about dialysis, what it does to you and how it helps you—I feel a little bit more comfortable now that I know much more.” (20s, in-center HD)*
**Challenges with life while receiving dialysis**	*“I miss traveling. That’s the biggest impairment to being on dialysis.” (70s, in-center HD)* *My husband and I like to travel. We can’t do that anymore. That’s why we take these little one day overnight things. (70s, in-center HD)*	*“I just only one wish to travel a lot but we don’t. That’s okay. I feel bad for my wife cannot go. I told her go alone but then oh, we have to go together.” (Male, 60s, in-center HD)* *“If you consider travel time down here, time for set up, time for take down, plus the runs. You’re looking at almost 15 hours in a week. That’s a part-time job. Yeah, it’s just a real pain in the rear. Biggest limitations I’d say is the diet and travel.” (30s, in-center HD)*

Abbreviation: HD, hemodialysis.

#### Mindset

In this study, mindset refers to a patient’s belief about their ability to respond to illness. Low regret participants expressed positivity in their quotes and painted a lighter picture of their illness course; they seemed to no longer be in conflict with dialysis. In contrast, those in the high regret group indicated they felt resigned to their fate. The mindset of patients influences their health behaviors and outcomes and shapes how they perceive their illness. It provides us with valuable insights into how patients view and approach their condition. Patients in the low regret group, with their positive mindset, are able to see their illness in a more empowering light, enabling them to navigate through challenges and find ways to thrive. On contrary, the high regret group’s mindset reflects the dichotomous choice they face, in which dialysis is seen as a last resort.

#### Reconciling Life While Receiving Dialysis

Patients’ adjustment to life while receiving dialysis was evident in their coping strategies, which is distinct from the theme of mindset. Although mindset refers to patients’ thoughts, beliefs, and perceptions about their illness, coping strategies involve the actions and behaviors they employ to adapt to life with dialysis. Significant differences in coping strategies were observed between the low regret and high regret groups, with the former employing more positive coping mechanisms. The low regret patients continued to have important goals for their lives despite dialysis. They were able to leverage their social support and demonstrated resilience by continuing to move forward. The high regret patients framed dialysis as an obligation for a variety of reasons, such as to stay alive, a lack of other options or a duty to others in their social circle. These differences in coping strategies highlight the diverse approaches patients take in adapting to life while receiving dialysis, with the low regret group demonstrating a more positive outlook and proactive engagement, while the high regret group viewed dialysis as a necessity with limited alternatives. Furthermore, coping strategies serve as an indicator of the contrasting approaches taken by the 2 groups of patients in actively responding to and reconciling with the challenges posed by their illness.

#### Biographic Disruption

Biographic disruption occurs when a chronic disease disrupts various aspects of a person’s life, such as their sense of identity, future plans, and interpersonal relationships, resulting in significant changes to their biography and social roles. It highlights the transformative impact of illness on individuals, altering their narratives and requiring them to adapt and navigate new circumstances.^23^ Distinct patterns of biographic disruption can be observed between patients with high regret and low regret. Those in the high regret group reported that dialysis had a detrimental effect on various aspects of their lives, including their ambitions, quality time with family, and significant life events. Patients expressed a profound sense of interruption in their very identity, with the emergence of a new identity shaped by their disease. Low regret patients experienced physical disruptions as well caused by the treatment, did not express the same sentiment. The subjective perception of disruption varied between the groups, indicating that the experience and expression of biographic disruption differed between individuals in the 2 groups.

Furthermore, high regret patients reported losing social connections and time with loved ones and expressed an inability to nurture relationships, missing activities that mattered to them, and distress about being a burden on others. Patients in the low regret group shared strategies on how they navigate and overcome barriers to engage in important life activities. The utilization of social support played a role in assisting patients in overcoming the challenges of biographic disruption.

#### Understanding and Learning Treatment Requirements

We observed differing levels of illness learning and understanding between the 2 groups. Both groups acknowledged the importance of time in comprehending the dialysis process, highlighting the significance of learning from fellow dialysis patients. In addition, experiential learning through engaging in dialysis was considered the most effective method of gaining knowledge. Despite these similarities, there were notable differences between both groups. High regret patients tended to engage in self-blame when faced with challenges in understanding their illness and its management. Conversely, low regret patients exhibited more self-compassion and were inclined to generalize their difficulties, recognizing them as expected and shared by others in similar circumstances.

#### Challenges With Life While Receiving Dialysis

We observed that some challenges were experienced equally between groups such as restricted travel and dietary changes. Travel is related to patient’s ambitions and biographic disruption. For example, a musician could not perform and had to give up playing because they could not travel. However, certain patients in both groups managed to adapt to short trips, although they noted that it still imposed limitations on their freedom and necessitated a substantial investment of time and effort. Those undergoing home dialysis were able to capitalize on this flexibility, enabling them to engage in more travel opportunities.

## Discussion

Previous research shows that decisional regret is associated with less patient autonomy, suboptimal education and planning regarding prognosis and life while receiving dialysis.^11^, ^12^, ^13^, ^14^, ^15^ In the only study using the DRS specifically, investigators found low regret in ∼8% of patients receiving dialysis in Singapore,^14^ similar to our low levels of regret overall. However, we observed that patients in both groups were equally informed about a discussion about prognosis. Despite our expectations that illness intrusiveness, a measure of the impact of kidney failure on one’s life, would be associated with decisional regret, this proved untrue (Table S2). Previous studies have demonstrated that illness intrusiveness is correlated with lower quality of life and greater symptom burden, such as pain, fatigue, anxiety, and depression.^24^ This finding may be because regret reflects the patient’s remorse for their current choice given how they are able to handle their perceived burden. It is plausible that someone who has a high illness intrusiveness could have low regret, should they have appropriate support of their capacity to cope with those burdens.^25^ However, high illness intrusiveness has also been correlated with lower patient capacity,^26^ so this relationship may deserve further quantitative inquiry.

Mindset plays a significant role in influencing health behaviors and outcomes.^27^, ^28^, ^29^, ^30^, ^31^ The development of an acceptance and growth mindset toward illness is a dynamic process, characterized by patients alternating between prioritizing health and life challenges until they achieve a balance.^31^, ^32^, ^33^ Integrating the biopsychosocial model and addressing mindset can be beneficial in enhancing the patient experience. Gaining an understanding of patients’ mindset provides valuable insights into their perspectives and can inform interventions aimed at fostering acceptance, growth, and a balanced outlook on their illness.^34^, ^35^, ^36^, ^37^ Health care providers can potentially play a role in shaping the mindset of patients undergoing dialysis by considering cultural, religious, media, social network, and trusted individuals’ influences, ultimately improving the patient experience.^38^, ^39^, ^40^

Patients expressing low regret opted for healthier coping mechanisms, such as leveraging social support, avoiding self-blame, engaging actively, and using adaptive coping strategies. Coping strategies used by patients with chronic illness including kidney disease have been described,^41^, ^42^, ^43^, ^44^, ^45^ with known coping strategies that have been beneficial for patient experience.^46^^,^^47^ Of importance, patients’ choice of coping strategies can change over time, influenced by their personal experience, social support system, individual beliefs, and availability of resources.^48^^,^^49^ The potential benefits of interventions aimed at promoting the use of healthy coping strategies among patients with kidney failure warrant further investigation.^49^, ^50^, ^51^, ^52^, ^53^ In existing literature, there are instances of studies emphasizing coping strategies beneficial for patients. For instance, one research study suggests that optimism indirectly affects health outcomes by promoting healthy behaviors, adaptive coping techniques, and enhancing positive mood. In addition, spirituality or religiosity may also play a role, particularly in certain populations.^41^^,^^54^^,^^55^

In our cohort, we observed that patients with low regret demonstrated strategies for resolving each stage of disruption, exhibited a capacity for bouncing back and maintained their pretreatment identities. Assisting patients in understanding and overcoming the disruptive process can significantly enhance patient-centered care.^56^^,^^57^ Furthermore, although not statistically significant, in our study patients may regret the treatment choice less with home dialysis modality, perhaps because of the preservation of some normalcy. This possibility should be examined further in future studies.

Finally, although patients from both groups acknowledged the challenges of navigating kidney disease and expressed the difficulty in fully comprehending the treatment process, patients with low regret demonstrated self-compassion and patients with high regret felt a sense of shame. This highlights the possible role of guidance from peers receiving dialysis about navigating this process. Patient narratives shared by peers can provide valuable support in coping with their illness and assist them in making informed health care decisions.^58^^,^^59^

In summary, our study indicates practical opportunities for interventions, such as addressing patients’ mindset and providing coaching on coping mechanisms, which may aid patients in navigating challenges more effectively. When preparing patients for kidney replacement therapy, early consideration of the workload capacity balance, incorporating patient-specific contextual information, and facilitating discussions with peers about different therapy options may contribute to minimizing disruption, improving understanding, and enhancing the overall patient experience.^60^, ^61^, ^62^, ^63^ Interventions that engage and activate patients may enhance their treatment satisfaction and decision-making process.^64^ Finally, it is important to acknowledge the significant overlap observed among the themes. This overlap could potentially be attributed to the fact that the emerging themes exist on a spectrum of related constructs, including patients’ acceptance, coping strategies, response to illness, and adaptation to disruptions.

Our study contributes to the existing knowledge by providing a comprehensive analysis of decisional regret among patients living with dialysis. With a dialysis vintage of less than 3 years, patients with absolute decisional regret may have withdrawn from dialysis^65^^,^^66^ and may not have been captured in our study. Decisional regret scale has limitations in quantifying extent of regret accurately, and regret is considered as a continuous variable rather than categorical. Therefore, we also chose to sample patients who answered, “I wish I had never started dialysis” to the question “How do you currently feel about starting dialysis to include patients with partial regret?” The emotion of regret in general, may signify a process of reflection and can change over time and is not always a negative emotion.^67^ However, in patients living with kidney failure, decisional regret affects their experience and satisfaction therefore, it is important to address.^68^^,^^69^ Surveying and interviewing patients in center has advantages and disadvantages. In the case of surveys, it provides the opportunity to help participants understand and correct doubts about survey questions, but some patients may not be comfortable expressing regret. Similarly, patients were offered the option of private clinical space or home interviews, but not all patients chose this. This influenced our decision to consider minimal signal of regret based on DRS score as evidence of regret.

In conclusion, the differences observed between the low regret and high regret groups highlight contrasting perspectives and responses to challenges. These emerging themes indicate potential psychologic and cognitive differences in how patients perceive and adjust to life while receiving dialysis, providing valuable insights into the complex factors that contribute to regret following the initiation of treatment.

## Supplementary Materials

Supplementary File (PDF) Figure S1; Item S1-S2; Table S1-S2.
