# Supplementary material for: Decisional Regret Surrounding Dialysis Initiation: A Comparative Analysis
Source: Kidney Med. 2023 Dec 20;6(3):100785. doi: 10.1016/j.xkme.2023.100785 (PMC10907211; doi:10.1016/j.xkme.2023.100785)
Supplement: Supplementary File (PDF) — Figure S1; Item S1-S2; Table S1-S2. [file mmc1.pdf]

Figure S1. Study Flowchart

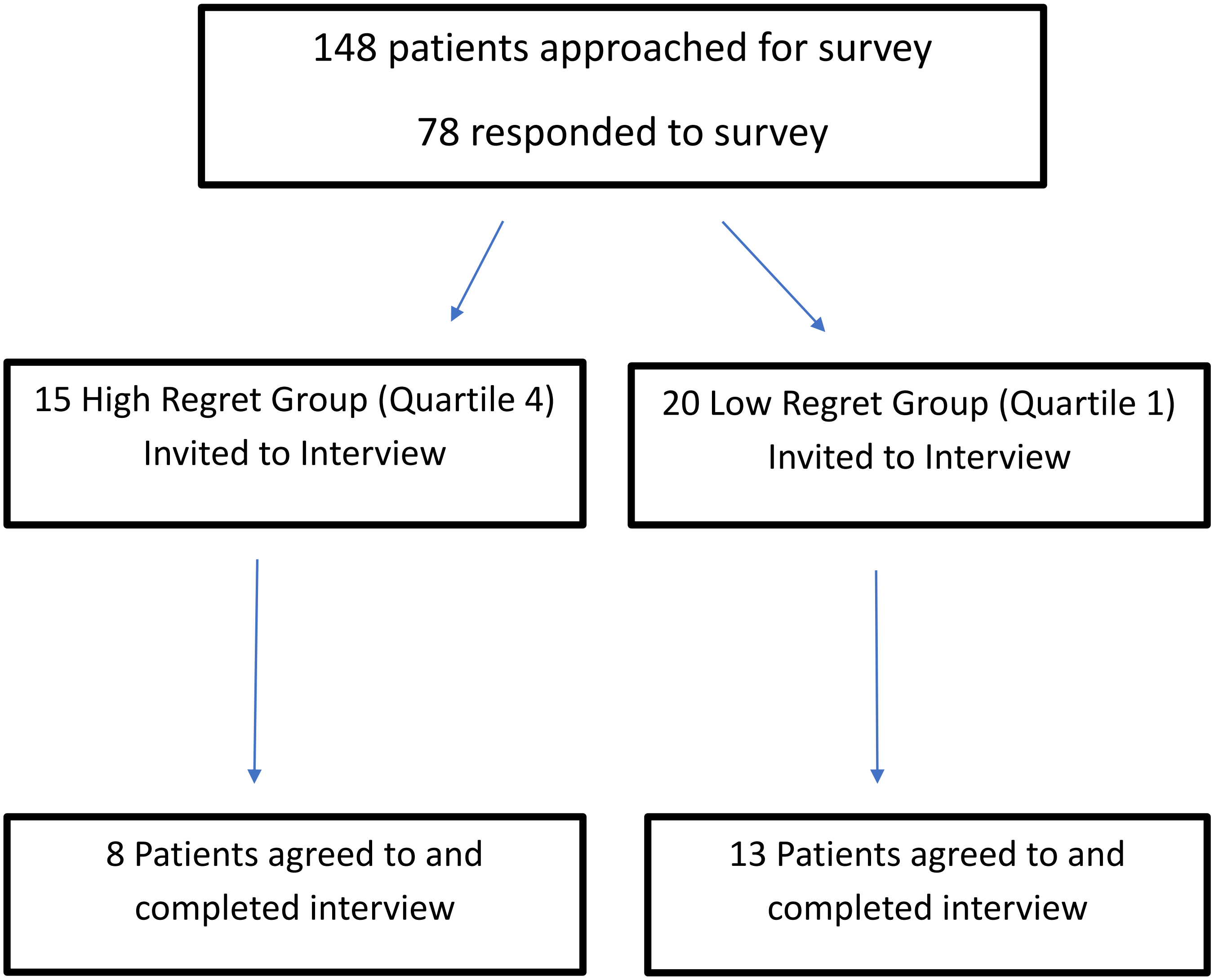

Item S1. Quantitative Survey

Today's Date: _____ / _____ / __________

Thank you for helping with this study. Your answers are important to us. Please take the time to read and answer each question carefully by marking the box that best represents your answer. Some items ask you to fill in the blank space provided for your answer. Your responses are confidential. After completing this survey, please return it to the clinical staff that gave it to you. Please feel free to ask any questions you might have while completing the survey.

1. How would you describe your current health?

1☐ Excellent
2☐ Very good
3☐ Good
4☐ Fair
5☐ Poor

Please answer the following questions regarding your kidney disease and starting dialysis.

2. How long have you received dialysis? __________ months / years (circle one)

3. Was the decision to begin dialysis:

1☐ Planned
2☐ Unplanned

4. Did you begin dialysis:

1☐ Inpatient / hospital setting
2☐ Outpatient / clinic setting

5. Are you on a kidney transplant list?

1☐ Yes
2☐ No
3☐ Choose not to answer

6. Which of the following options were presented to you as treatment options to manage your renal failure? (Check all that apply.)

1☐ Peritoneal dialysis
2☐ Home hemodialysis
3☐ Supportive care without dialysis
4☐ Renal transplant
5☐ None of the above

6☐ Choose not to answer

7. Who mostly influenced your decision to start dialysis?

1☐ Primary care provider
2☐ Nephrology provider
3☐ Family
4☐ Myself
5☐ Other, please specify: ______________________________
6☐ Choose not to answer

8. How do you currently feel about starting dialysis?

1☐ Best decision I have made
2☐ Not as bad as I thought it would be
3☐ I thought it would be better, but I am okay with it
4☐ I wish I had never started dialysis
5☐ Other, please specify: ______________________________
6☐ Choose not to answer

9. I feel well prepared for what to expect with dialysis.

1☐ Strongly agree
2☐ Agree
3☐ Undecided
4☐ Disagree
5☐ Strongly disagree
6☐ Choose not to answer

10. How did your loved ones feel about your decision?

1☐ Highly satisfied
2☐ Satisfied
3☐ Unhappy
4☐ Very unhappy
5☐ Uncertain
6☐ Choose not to answer

11. The cost of dialysis played a role in my decision to start dialysis.

1☐ Strongly agree
2☐ Agree
3☐ Undecided
4☐ Disagree
5☐ Strongly disagree
6☐ Choose not to answer

12. Were the out-of-pocket costs of dialysis discussed prior to starting treatment?

1☐ Yes
2☐ No
3☐ Choose not to answer

13. Were the needs associated with dialysis (e.g., self-care, coming to appointments, etc.) discussed prior to starting treatment?

1☐ Yes
2☐ No

**Please answer the following questions about prognosis (life expectancy).**

14. Has anyone on your care team specifically discussed your prognosis (life expectancy) with you?

1☐ Yes
2☐ No
3☐ Choose not to answer

15. How well informed do you feel about your current prognosis (life expectancy)?

1☐ Very well informed
2☐ Somewhat informed
3☐ Unsure
4☐ Somewhat uninformed
5☐ Completely uninformed
6☐ Choose not to answer

16. How do you expect your health to be in 12 months from now?

1☐ Much better
2☐ Somewhat better
3☐ The same
4☐ Worse
5☐ Much worse
6☐ Choose not to answer

17. If you were seriously ill, would you prefer care to:

1☐ Extend life, even if it meant more pain and discomfort
2☐ Relieve pain and discomfort, even if it meant not living as long

Please fill out the following questions related to your kidney disease and the dialysis treatments you use to manage it.

18. How much do your illness and/or its treatment interfere with…

your feeling of being healthy?

0☐	1☐	2☐	3☐	4☐	5☐	6☐	7☐
Not applicable	Not very much						Very much

the things you eat and drink?

0☐	1☐	2☐	3☐	4☐	5☐	6☐	7☐
Not applicable	Not very much						Very much

your work, including job, house work, chores, or errands?

0☐	1☐	2☐	3☐	4☐	5☐	6☐	7☐
Not applicable	Not very much						Very much

playing sports, gardening, or other physical recreation or hobbies?

0☐	1☐	2☐	3☐	4☐	5☐	6☐	7☐
Not applicable	Not very much						Very much

quiet recreation or hobbies, such as reading, TV, music, knitting, etc.?

0☐	1☐	2☐	3☐	4☐	5☐	6☐	7☐
Not applicable	Not very much						Very much

your financial situation?

0☐	1☐	2☐	3☐	4☐	5☐	6☐	7☐
Not applicable	Not very much						Very much

your relationship with your spouse or domestic partner?

0☐	1☐	2☐	3☐	4☐	5☐	6☐	7☐
Not applicable	Not very much						Very much

19. How much do your illness and/or its treatment interfere with…

your sex life?

0☐	1☐	2☐	3☐	4☐	5☐	6☐	7☐
Not applicable	Not very much						Very much

your relationship and social activities with your family?

0☐	1☐	2☐	3☐	4☐	5☐	6☐	7☐
Not applicable	Not very much						Very much

social activities with your friends, neighbors, or groups?

0☐	1☐	2☐	3☐	4☐	5☐	6☐	7☐
Not applicable	Not very much						Very much

your religious or spiritual activities?

0☐	1☐	2☐	3☐	4☐	5☐	6☐	7☐
Not applicable	Not very much						Very much

your involvement in community or civic activities?

0☐	1☐	2☐	3☐	4☐	5☐	6☐	7☐
Not applicable	Not very much						Very much

your self-improvement or self-expression activities?

0☐	1☐	2☐	3☐	4☐	5☐	6☐	7☐
Not applicable	Not very much						Very much

Please think about the decision you made about starting dialysis to treat your kidney disease. Please show how you feel about these statements by selecting a response from Strongly Agree to Strongly Disagree.

	Strongly Agree	Agree	Neither Agree nor Disagree	Disagree	Strongly Disagree
20. It was the right decision	1☐	2☐	3☐	4☐	5☐
21. I regret the choice that was made	1☐	2☐	3☐	4☐	5☐
22. I would go for the same choice if I had to do it over again	1☐	2☐	3☐	4☐	5☐
23. The choice did me a lot of harm	1☐	2☐	3☐	4☐	5☐
24. The decision was a wise one	1☐	2☐	3☐	4☐	5☐

Item S2. Qualitative Interview Guide

Participant #_______________

Date_______/_____/_______

Interview Guide

Hi, thank you for coming in today. My name is [researcher name]. I am a researcher here, at Mayo Clinic. We are doing this study because we want to learn more about what your life on dialysis is like.

This interview will take about 60 minutes. The interview will focus on your personal experience of managing kidney disease and dialysis treatment.

I will ask you a series of questions which follow a loose structure, and I will ask you follow up questions based on your answers. As an interviewer, my job is to learn about how you live with dialysis in a lot of detail, so I will ask questions that may at times seem boring or overly detailed but actually help us to understand your life and experiences in a lot of depth. We can veer away from the set of questions I have in front of me but I may at times bring us back to this set of questions to make sure I get all of the information I need. Also, please be aware that i will try to stay neutral during this process so it may feel a little more awkward than your average back and forth conversation. I may take notes while you talk, but even if I'm not making eye contact I am listening to you.

Do I have your permission to voice record this interview so that later, I can go back and make sure I transcribe this conversation accurately? Ok, I will put this recorded somewhere where it will pick up both of our voices. I'll check it from time to time to make sure it is recording but we can mostly forget about it.

Everything you say in our interview is confidential. I am part of an independent research group at Mayo Clinic, and not a part of the clinical care team. You can feel free to share your honest opinions with me, and I will not directly share them with the people who provide your care. The only reason we would inform your clinical care team of anything would be if we believe you could be at risk of harming yourself or those you love.

Though quotes from our conversations may appear in research publications or be used in academic workshops or presentations, your name will not be linked to this information. We may change

details that don't matter very much to the meaning of your words in order to further keep your identity private. If at any time you feel uncomfortable answering a question and would like to skip it, would like to stop the use of the recorder or the interview itself, please let me know. I am very interested in your day-to-day experience.

INTERVIEW GUIDE

THESE QUESTIONS ARE A SEMI-STRUCTURED GUIDE OF WHAT WILL BE DISCUSSED WITH PATIENTS AND MAY BE REVISED SLIGHTLY MODIFIED AS INTERVIEWS PROGRESS AND WE LEARN NEW INFORMATION FROM OUR PARTICIPANTS.

The overarching reason for talking with you today is to understand what your experience on dialysis is like, what you do to manage your health while on dialysis, and your feelings about starting dialysis.

1. Tell me how things have been going in managing your health lately?
    a. *What other conditions are you managing in addition to your Kidney Disease?*

2. Would you mind just walking me through what the process looked like when you began dialysis from the moment someone mentioned to you that you might need or would be starting dialysis?
    a. Probes:
        i. *What was going on in your life during that time?*
        ii. *What was going on with your health during that time?*
        iii. *How was the potential need to go on dialysis brought up to you?*
        iv. *Who was there supporting you during that time?*
            1. *What role did each person play during your decision making process?*

v. *What were your feelings about starting dialysis before it began?*

3. I want to understand your feelings about dialysis now that you have been on it for some time.
    a. Can you tell me about how you feel now about dialysis, compared to before you began dialysis?
    b. Probes:
        i. *What makes you feel that way?*
        ii. *What, if any, are the things you wish you would have known before beginning?*

4. I'd like to understand what your day-to-day activities of managing dialysis are. The next two questions will ask for you to give us a detailed account of what you do on dialysis and non-dialysis days.
    a. What does your typical full day look like on your dialysis days? Start with what you do when you first wake up in the morning and go from there.
        i. PROMPT: What next?
        ii. PROMPT: Can you give me more detail on that?
            1. *Activities to ask them about-- check items off as they come up in the interview ASK FOR DETAIL*
                a. *Grocery shopping* ☐
                b. *Planning meals* ☐
                c. *Food preparation/eating* ☐
                d. *Taking medication* ☐
                e. *Organizing/managing medication* ☐
                f. *scheduling* ☐
                    i. *related to medical needs* ☐
                    ii. *related to other (work ☐ social ☐ family needs ☐)*
                g. *transportation to the clinic* ☐
                h. *setting up dialysis (for at-home)* ☐
                i. *coordinating with support person(s)* ☐
                j. *for participants who work: carrying out work activities* ☐
                k. *managing bodily symptoms (e.g. fatigue, cramps, nausea, limited freedom of movement, headache)* ☐
                l. *planning ahead for sitting during tx* ☐
                m. *passing time during tx (e.g. sleep, watch TV, read)* ☐

    b. What is your perspective on how manageable these specific activities are that are involved in your care?
        i. Probe
            1. *Are there certain activities that are easier?*
                a. *What makes you think/say that?*
            2. *Are there certain activities that you struggle with more than others?*
                a. *What makes you think/say that?*

c. What does your typical full day look like on your non-dialysis days? Start with what you do when you first wake up and so on
    i. PROMPTS: What next?
    ii. PROMPTS: Will you tell me more detail
        1. *Activities to ask them about-- check items off as they come up in the interview and ASK FOR DETAIL*
            a. *Grocery shopping* ☐
            b. *Planning meals* ☐
            c. *Food preparation/eating* ☐
            d. *Taking medication* ☐
            e. *Organizing/managing medication* ☐
            f. *scheduling* ☐
                i. *related to medical needs* ☐
                ii. *related to other (work* ☐ *social* ☐ *family needs* ☐*)*
            g. *transportation to the clinic* ☐
            h. *setting up dialysis (for at-home)* ☐
            i. *coordinating with support person(s)* ☐
            j. *for participants who work: carrying out work activities* ☐
            k. *managing bodily symptoms (e.g. fatigue, cramps, nausea, limited freedom of movement, headache)* ☐

5. Who participates in daily activities with you?
    a. *Activities to ask them about-- check items off as they come up in the interview and ASK FOR DETAIL*
        i. *Specific people they name (what kind of support do these people provide, emotional or instrumental)* ☐
        ii. *Are there people you used to spend more time with before you were on dialysis?* ☐
    b. Probes
        i. Those facilitating emotional//social support
        ii. Those facilitating instrumental social support

6. Tell me about how have your daily activities now have changed from your daily activities before you began dialysis?
    a. Probes
        i. *Working* ☐
        ii. *Volunteering* ☐
        iii. *Hobbies* ☐
        iv. *Caregiving* ☐
        v. *Social activities* ☐

7. Tell me about the people who help you with your care at the clinic?
    a. Probes:

i. *What specific activities that these individuals do that are helpful*
ii. *What do you find frustrating when interacting with your care or the health care environment in general?*

The next questions deal with insurance, billing, and payment.

8. Can you tell me about your experiences dealing with managing insurance, billing, and payment related to kidney disease, dialysis, and related health conditions (refer to co-morbid conditions they described earlier)?

9. What specific problems have you had with insurance, billing, and payment?
    a. *PROMPT: Think about an issue that you have had with insurance and/or billing/payment and describe it to me in detail?*

10. How do you manage and organize the billing and insurance aspect of your care?
    a. *PROMPT: Do you or your caregiver have any specific routines or organizational tools that you use to deal with insurance, billing, and payment?*
        i. *Check off topics as they are discussed*
            1. *Managing insurance* ☐
            2. *Managing billing* ☐
            3. *Organizing mail/paper documentation* ☐
            4. *online payment/mail payments* ☐

The next questions deal with feeling and perspectives of living on dialysis

******If patient is expressing they feel no joy in their life, locate most trusted Mayo clinician to reach out to inform them of depression/suicidal-related concern about said patient*******

11. Where do you find the most joy in your life?
    a. *Activities to ask them about-- check items off as they come up in the interview and ASK FOR DETAIL*
        i. *In what ways has dialysis helped you maintain {this joyful activity}?*
        ii. *In what ways has dialysis hindered you in keeping up with {this joyful activity}?*
    b. *Would your answer to this question have been different before you began dialysis?*
        i. *If yes, how so?*
        ii. *e) If no, proceed to question #*

If time, ask remaining questions

12. In what ways do you manage and/or maintain the things that bring you joy and happiness
    a. *Interpersonal connections (family, friends)*
    b. *Spiritual groups*
    c. *Journaling*
    d. *Support groups*

13. What do you find are the most problematic parts of being on dialysis?
    a. *How have these changed over time?*
    b. *Did these meet your expectations of the problematic pieces?*

14. What do you find are the least problematic parts of being on dialysis?
    a. *How have these changed over time?*
    b. *Did these meet your expectations of the least problematic pieces?*

The next questions are more specifically about the process of starting dialysis specifically, so I will ask you reflect about the past as well as the present.

15. What types of information were you given just before or at the time you began dialysis?
    a. *Information about cost, about frequency, about lifestyle requirements, etc.*

16. In what ways was that information helpful in beginning dialysis?

17. In what ways was that information not helpful, frustrating, or overwhelming in beginning dialysis?

18. Choices related to dialysis treatment could be: about when to begin dialysis, different types of dialysis, or to begin versus not begin dialysis. Did someone mention to you any of these types of choices during the time of starting dialysis?

➔ *If yes, which choices were mentioned? Can you tell me more about them?*
➔ *If no, thank patient for answering and proceed to question 8.*

19. Who was it that offered those choices and what did your conversation with that person look like?
    a. *What were your feelings about those choices at the time?*
    b. *What are your feelings about those choices now?*

20. Who did you involve in those choices?
    a. *Tell me more about that person's role in the conversation or in your decisions related to dialysis treatment.*

21. I want to understand your feelings about dialysis now that you have been on it for some time. Can you tell me about how you feel now about dialysis?

22. How does that compare to how you felt before you started?
    a. *What makes you feel that way?*
    b. *What, if any, are the things you wish you would have known before beginning?*

23. What would you like other people potentially facing a dialysis start to know before they begin?

24. What else would you like share about your feelings about starting dialysis or being on dialysis?

Table S1. Participant characteristics and survey responses for all DRS quartiles

	DRS Quartiles				
	Quartile 1: 0 (N=20)	Quartile 2: 1-10 (N=20)	Quartile 3: 11-25 (N=17)	Quartile 4: >25 (N=15)	Total (N=72)
**Age:**					
N	20	20	17	15	72
Median (IQR)	63.5 (49.5, 70.0)	56.0 (47.0, 76.5)	72.0 (47.0, 79.0)	60.0 (36.0, 73.0)	62.5 (47.0, 75.0)
**Sex:**, n (%)					
Female	10 (50.0%)	5 (25.0%)	6 (35.3%)	5 (33.3%)	26 (36.1%)
Male	10 (50.0%)	15 (75.0%)	11 (64.7%)	10 (66.7%)	46 (63.9%)
**Race:**, n (%)					
White	18 (90.0%)	16 (80.0%)	14 (87.5%)	8 (57.1%)	56 (80.0%)
Black or African American	0 (0.0%)	1 (5.0%)	0 (0.0%)	4 (28.6%)	5 (7.1%)
Asian	0 (0.0%)	2 (10.0%)	2 (12.5%)	1 (7.1%)	5 (7.1%)
Other	2 (10.0%)	1 (5.0%)	0 (0.0%)	1 (7.1%)	4 (5.7%)
Missing	0	0	1	1	2
**Ethnicity:**, n (%)					
Hispanic or Latino	1 (5.0%)	0 (0.0%)	1 (5.9%)	1 (6.7%)	3 (4.2%)
Not Hispanic or Latino	19 (95.0%)	20 (100.0%)	15 (88.2%)	14 (93.3%)	68 (94.4%)
Choose Not to Disclose	0 (0.0%)	0 (0.0%)	1 (5.9%)	0 (0.0%)	1 (1.4%)
**1. How would you describe your current health?**, n (%)					
Excellent	1 (5.0%)	0 (0.0%)	0 (0.0%)	2 (13.3%)	3 (4.2%)
Good	7 (35.0%)	9 (45.0%)	7 (41.2%)	5 (33.3%)	28 (38.9%)
Fair	5 (25.0%)	4 (20.0%)	6 (35.3%)	6 (40.0%)	21 (29.2%)
Very good	3 (15.0%)	4 (20.0%)	4 (23.5%)	2 (13.3%)	13 (18.1%)
Poor	4 (20.0%)	3 (15.0%)	0 (0.0%)	0 (0.0%)	7 (9.7%)
**How long have you received dialysis (years)?**					
N	20	19	17	13	69
Median (IQR)	3.5 (1.1, 5.8)	2.2 (1.0, 5.0)	2.5 (1.0, 3.2)	2.0 (1.0, 5.0)	3.0 (1.0, 5.0)
**Was the decision to begin dialysis:**, n (%)					
Planned	9 (45.0%)	12 (63.2%)	10 (58.8%)	7 (50.0%)	38 (54.3%)
Unplanned	11 (55.0%)	7 (36.8%)	7 (41.2%)	7 (50.0%)	32 (45.7%)
Missing	0	1	0	1	2
**4. Did you begin dialysis:**, n (%)					
Inpatient / hospital setting	8 (40.0%)	7 (35.0%)	7 (41.2%)	7 (46.7%)	29 (40.3%)
Outpatient / clinic setting	12 (60.0%)	13 (65.0%)	10 (58.8%)	8 (53.3%)	43 (59.7%)
**Are you on a kidney transplant list?**, n (%)					
No	14 (70.0%)	12 (60.0%)	13 (76.5%)	9 (64.3%)	48 (67.6%)
Yes	6 (30.0%)	8 (40.0%)	4 (23.5%)	5 (35.7%)	23 (32.4%)
Missing	0	0	0	1	1

	DRS Quartiles				
	Quartile 1: 0 (N=20)	Quartile 2: 1-10 (N=20)	Quartile 3: 11-25 (N=17)	Quartile 4: >25 (N=15)	Total (N=72)
**6. Which of the following options were presented to you as treatment options to manage your renal failure? (Check all that apply.) (choice=Peritoneal dialysis)**, n (%)					
Unchecked	7 (35.0%)	9 (45.0%)	6 (35.3%)	5 (33.3%)	27 (37.5%)
Checked	13 (65.0%)	11 (55.0%)	11 (64.7%)	10 (66.7%)	45 (62.5%)
**6. Which of the following options were presented to you as treatment options to manage your renal failure? (Check all that apply.) (choice=Home hemodialysis)**, n (%)					
Unchecked	4 (20.0%)	9 (45.0%)	5 (29.4%)	12 (80.0%)	30 (41.7%)
Checked	16 (80.0%)	11 (55.0%)	12 (70.6%)	3 (20.0%)	42 (58.3%)
**6. Which of the following options were presented to you as treatment options to manage your renal failure? (Check all that apply.) (choice=Supportive care without dialysis)**, n (%)					
Unchecked	14 (70.0%)	17 (85.0%)	12 (70.6%)	14 (93.3%)	57 (79.2%)
Checked	6 (30.0%)	3 (15.0%)	5 (29.4%)	1 (6.7%)	15 (20.8%)
**6. Which of the following options were presented to you as treatment options to manage your renal failure? (Check all that apply.) (choice=Renal transplant)**, n (%)					
Unchecked	9 (45.0%)	10 (50.0%)	10 (58.8%)	9 (60.0%)	38 (52.8%)
Checked	11 (55.0%)	10 (50.0%)	7 (41.2%)	6 (40.0%)	34 (47.2%)
**6. Which of the following options were presented to you as treatment options to manage your renal failure? (Check all that apply.) (choice=None of the above)**, n (%)					
Unchecked	18 (90.0%)	18 (90.0%)	15 (88.2%)	14 (93.3%)	65 (90.3%)
Checked	2 (10.0%)	2 (10.0%)	2 (11.8%)	1 (6.7%)	7 (9.7%)
**6. Which of the following options were presented to you as treatment options to manage your renal failure? (Check all that apply.) (choice=Choose not to answer)**, n (%)					
Unchecked	20 (100.0%)	18 (90.0%)	17 (100.0%)	15 (100.0%)	70 (97.2%)
Checked	0 (0.0%)	2 (10.0%)	0 (0.0%)	0 (0.0%)	2 (2.8%)
**7. Who mostly influenced your decision to start dialysis? (choice=Primary care provider)**, n (%)					
Unchecked	18 (90.0%)	16 (80.0%)	14 (82.4%)	12 (80.0%)	60 (83.3%)
Checked	2 (10.0%)	4 (20.0%)	3 (17.6%)	3 (20.0%)	12 (16.7%)

	DRS Quartiles				
	Quartile 1: 0 (N=20)	Quartile 2: 1-10 (N=20)	Quartile 3: 11-25 (N=17)	Quartile 4: >25 (N=15)	Total (N=72)
**7. Who mostly influenced your decision to start dialysis? (choice=Nephrology provider)**, n (%)					
Unchecked	8 (40.0%)	9 (45.0%)	5 (29.4%)	6 (40.0%)	28 (38.9%)
Checked	12 (60.0%)	11 (55.0%)	12 (70.6%)	9 (60.0%)	44 (61.1%)
**7. Who mostly influenced your decision to start dialysis? (choice=Family)**, n (%)					
Unchecked	19 (95.0%)	18 (90.0%)	15 (88.2%)	11 (73.3%)	63 (87.5%)
Checked	1 (5.0%)	2 (10.0%)	2 (11.8%)	4 (26.7%)	9 (12.5%)
**7. Who mostly influenced your decision to start dialysis? (choice=Myself)**, n (%)					
Unchecked	16 (80.0%)	16 (80.0%)	13 (76.5%)	13 (86.7%)	58 (80.6%)
Checked	4 (20.0%)	4 (20.0%)	4 (23.5%)	2 (13.3%)	14 (19.4%)
**7. Who mostly influenced your decision to start dialysis? (choice=Other)**, n (%)					
Unchecked	18 (90.0%)	18 (90.0%)	16 (94.1%)	13 (86.7%)	65 (90.3%)
Checked	2 (10.0%)	2 (10.0%)	1 (5.9%)	2 (13.3%)	7 (9.7%)
**7. Who mostly influenced your decision to start dialysis? (choice=Choose not to answer)**, n (%)					
Unchecked	20 (100.0%)	19 (95.0%)	17 (100.0%)	15 (100.0%)	71 (98.6%)
Checked	0 (0.0%)	1 (5.0%)	0 (0.0%)	0 (0.0%)	1 (1.4%)
**7. (cont.) If Other, please specify:**, n (%)					
Death	1 (50.0%)	0 (0.0%)	0 (0.0%)	0 (0.0%)	1 (14.3%)
Doctors at the VA hospital	0 (0.0%)	0 (0.0%)	1 (100.0%)	0 (0.0%)	1 (14.3%)
LVAD Department	0 (0.0%)	1 (50.0%)	0 (0.0%)	0 (0.0%)	1 (14.3%)
Results of heart surgery - cardiac physician	0 (0.0%)	0 (0.0%)	0 (0.0%)	0 (0.0%)	0 (0.0%)
When I went to the ER and was told my numbers were bad and I was retaining fluid in my lungs.	0 (0.0%)	1 (50.0%)	0 (0.0%)	0 (0.0%)	1 (14.3%)
Wife	0 (0.0%)	0 (0.0%)	0 (0.0%)	1 (50.0%)	1 (14.3%)
friends	0 (0.0%)	0 (0.0%)	0 (0.0%)	1 (50.0%)	1 (14.3%)
life or death waiting for kidney	1 (50.0%)	0 (0.0%)	0 (0.0%)	0 (0.0%)	1 (14.3%)
Missing	18	18	16	13	65
**8. How do you currently feel about starting dialysis?**, n (%)					
Best decision I have made	4 (20.0%)	5 (26.3%)	3 (17.6%)	1 (7.7%)	13 (18.8%)
Not as bad as I thought it would be	8 (40.0%)	8 (42.1%)	11 (64.7%)	4 (30.8%)	31 (44.9%)
I thought it would be better, but I am okay with it	7 (35.0%)	1 (5.3%)	1 (5.9%)	5 (38.5%)	14 (20.3%)
I wish I had never started dialysis	1 (5.0%)	0 (0.0%)	1 (5.9%)	1 (7.7%)	3 (4.3%)
Other	0 (0.0%)	5 (26.3%)	1 (5.9%)	2 (15.4%)	8 (11.6%)
Missing	0	1	0	2	3

	DRS Quartiles				
	Quartile 1: 0 (N=20)	Quartile 2: 1-10 (N=20)	Quartile 3: 11-25 (N=17)	Quartile 4: >25 (N=15)	Total (N=72)
**8. (cont.) If Other, please specify:**, n (%)					
'If I didn't start, I'd die'	0 (%)	1 (20.0%)	0 (0.0%)	0 (0.0%)	1 (12.5%)
(Pt also marked 'i wish I had never started dialysis') 'Hate having to do it every night but I am glad to be for my wife and kids'	0 (%)	1 (20.0%)	0 (0.0%)	0 (0.0%)	1 (12.5%)
I just went along with the flow	0 (%)	1 (20.0%)	0 (0.0%)	0 (0.0%)	1 (12.5%)
I knew my third transplant was failing and needed to start dialysis soon. It was very difficult to start dialysis again.	0 (%)	0 (0.0%)	1 (100.0%)	0 (0.0%)	1 (12.5%)
I wish I had never started dialysis, but I had no option, I would die	0 (%)	1 (20.0%)	0 (0.0%)	0 (0.0%)	1 (12.5%)
I'm alive.....	0 (%)	0 (0.0%)	0 (0.0%)	1 (50.0%)	1 (12.5%)
In the beginning it was miserable, but now I can manage it and live with dialysis	0 (%)	1 (20.0%)	0 (0.0%)	0 (0.0%)	1 (12.5%)
It's okay, I will live with it	0 (%)	0 (0.0%)	0 (0.0%)	1 (50.0%)	1 (12.5%)
Missing	20	15	16	13	64
**9. I feel well prepared for what to expect with dialysis.**, n (%)					
Strongly agree	5 (25.0%)	6 (31.6%)	4 (23.5%)	3 (23.1%)	18 (26.1%)
Agree	12 (60.0%)	13 (68.4%)	10 (58.8%)	4 (30.8%)	39 (56.5%)
Undecided	1 (5.0%)	0 (0.0%)	2 (11.8%)	6 (46.2%)	9 (13.0%)
Strongly disagree	2 (10.0%)	0 (0.0%)	1 (5.9%)	0 (0.0%)	3 (4.3%)
Missing	0	1	0	2	3
**10. How did your loved ones feel about your decision?**, n (%)					
Highly satisfied	10 (50.0%)	7 (36.8%)	7 (41.2%)	1 (7.7%)	25 (36.2%)
Satisfied	9 (45.0%)	11 (57.9%)	9 (52.9%)	9 (69.2%)	38 (55.1%)
Unhappy	0 (0.0%)	0 (0.0%)	1 (5.9%)	0 (0.0%)	1 (1.4%)
Very unhappy	0 (0.0%)	0 (0.0%)	0 (0.0%)	1 (7.7%)	1 (1.4%)
Uncertain	1 (5.0%)	1 (5.3%)	0 (0.0%)	2 (15.4%)	4 (5.8%)
Missing	0	1	0	2	3
**11. The cost of dialysis played a role in my decision to start dialysis.**, n (%)					
Strongly agree	1 (5.0%)	0 (0.0%)	0 (0.0%)	1 (8.3%)	2 (3.0%)
Agree	3 (15.0%)	6 (30.0%)	2 (13.3%)	3 (25.0%)	14 (20.9%)
Undecided	1 (5.0%)	4 (20.0%)	1 (6.7%)	4 (33.3%)	10 (14.9%)
Disagree	4 (20.0%)	7 (35.0%)	6 (40.0%)	3 (25.0%)	20 (29.9%)
Strongly disagree	11 (55.0%)	3 (15.0%)	6 (40.0%)	1 (8.3%)	21 (31.3%)
Missing	0	0	2	3	5
**14. Has anyone on your care team specifically discussed your prognosis (life expectancy) with you?**, n (%)					
No	10 (50.0%)	9 (45.0%)	10 (58.8%)	8 (66.7%)	37 (53.6%)
Yes	10 (50.0%)	11 (55.0%)	7 (41.2%)	4 (33.3%)	32 (46.4%)
Missing	0	0	0	3	3

	DRS Quartiles				
	Quartile 1: 0 (N=20)	Quartile 2: 1-10 (N=20)	Quartile 3: 11-25 (N=17)	Quartile 4: >25 (N=15)	Total (N=72)
**15. How well informed do you feel about you current prognosis (life expectancy)?**, n (%)					
Very well informed	9 (45.0%)	6 (30.0%)	6 (35.3%)	2 (14.3%)	23 (32.4%)
Somewhat informed	6 (30.0%)	9 (45.0%)	5 (29.4%)	8 (57.1%)	28 (39.4%)
Unsure	4 (20.0%)	3 (15.0%)	4 (23.5%)	3 (21.4%)	14 (19.7%)
Somewhat uninformed	1 (5.0%)	0 (0.0%)	1 (5.9%)	1 (7.1%)	3 (4.2%)
Completely uninformed	0 (0.0%)	1 (5.0%)	1 (5.9%)	0 (0.0%)	2 (2.8%)
Choose not to answer	0 (0.0%)	1 (5.0%)	0 (0.0%)	0 (0.0%)	1 (1.4%)
Missing	0	0	0	1	1
**16. How do you expect your health to be in 12 months from now?**, n (%)					
Much better	4 (20.0%)	3 (15.8%)	3 (17.6%)	3 (23.1%)	13 (18.8%)
Somewhat better	3 (15.0%)	8 (42.1%)	4 (23.5%)	4 (30.8%)	19 (27.5%)
The same	10 (50.0%)	7 (36.8%)	10 (58.8%)	6 (46.2%)	33 (47.8%)
Worse	3 (15.0%)	1 (5.3%)	0 (0.0%)	0 (0.0%)	4 (5.8%)
Missing	0	1	0	2	3
**17. If you were seriously ill, would you prefer care to:**, n (%)					
Extend life, even if it meant more pain and discomfort	11 (61.1%)	6 (31.6%)	7 (43.8%)	8 (66.7%)	32 (49.2%)
Relieve pain and discomfort, even if it meant not living as long	7 (38.9%)	13 (68.4%)	9 (56.3%)	4 (33.3%)	33 (50.8%)
Missing	2	1	1	3	7
**20. It was the right decision**, n (%)					
Strongly Agree	20 (100.0%)	19 (95.0%)	6 (35.3%)	2 (13.3%)	47 (65.3%)
Agree	0 (0.0%)	1 (5.0%)	11 (64.7%)	7 (46.7%)	19 (26.4%)
Neither Agree nor Disagree	0 (0.0%)	0 (0.0%)	0 (0.0%)	5 (33.3%)	5 (6.9%)
Disagree	0 (0.0%)	0 (0.0%)	0 (0.0%)	1 (6.7%)	1 (1.4%)
**21. I regret the choice that was made**, n (%)					
Strongly Agree	0 (0.0%)	0 (0.0%)	1 (5.9%)	1 (6.7%)	2 (2.8%)
Agree	0 (0.0%)	0 (0.0%)	1 (5.9%)	3 (20.0%)	4 (5.6%)
Neither Agree nor Disagree	0 (0.0%)	1 (5.0%)	0 (0.0%)	8 (53.3%)	9 (12.5%)
Disagree	0 (0.0%)	9 (45.0%)	9 (52.9%)	3 (20.0%)	21 (29.2%)
Strongly Disagree	20 (100.0%)	10 (50.0%)	6 (35.3%)	0 (0.0%)	36 (50.0%)
**22. I would go for the same choice if I had to do it over again**, n (%)					
Strongly Agree	20 (100.0%)	17 (85.0%)	3 (17.6%)	3 (20.0%)	43 (59.7%)
Agree	0 (0.0%)	3 (15.0%)	10 (58.8%)	6 (40.0%)	19 (26.4%)
Neither Agree nor Disagree	0 (0.0%)	0 (0.0%)	1 (5.9%)	5 (33.3%)	6 (8.3%)
Disagree	0 (0.0%)	0 (0.0%)	0 (0.0%)	1 (6.7%)	1 (1.4%)
Strongly Disagree	0 (0.0%)	0 (0.0%)	3 (17.6%)	0 (0.0%)	3 (4.2%)
**23. The choice did me a lot of harm**, n (%)					
Strongly Agree	0 (0.0%)	0 (0.0%)	0 (0.0%)	3 (20.0%)	3 (4.2%)
Neither Agree nor Disagree	0 (0.0%)	3 (15.0%)	1 (5.9%)	5 (33.3%)	9 (12.5%)
Disagree	0 (0.0%)	8 (40.0%)	9 (52.9%)	7 (46.7%)	24 (33.3%)
Strongly Disagree	20 (100.0%)	9 (45.0%)	7 (41.2%)	0 (0.0%)	36 (50.0%)

	DRS Quartiles				
	Quartile 1: 0 (N=20)	Quartile 2: 1-10 (N=20)	Quartile 3: 11-25 (N=17)	Quartile 4: >25 (N=15)	Total (N=72)
**24. The decision was a wise one**, n (%)					
Strongly Agree	20 (100.0%)	17 (85.0%)	6 (35.3%)	2 (13.3%)	45 (62.5%)
Agree	0 (0.0%)	3 (15.0%)	10 (58.8%)	7 (46.7%)	20 (27.8%)
Neither Agree nor Disagree	0 (0.0%)	0 (0.0%)	0 (0.0%)	6 (40.0%)	6 (8.3%)
Strongly Disagree	0 (0.0%)	0 (0.0%)	1 (5.9%)	0 (0.0%)	1 (1.4%)

Table S2. **Participant characteristics and survey responses; comparing DRS quartiles 1 and 4**

	**DRS Quartiles**		
	**Quartile 1: 0 (N=20)**	**Quartile 4: >25 (N=15)**	**P-value**
**Age:**			0.79[1]
N	20	15	
Median (IQR)	63.5 (49.5, 70.0)	60.0 (36.0, 73.0)	
**Sex:**, n (%)			0.49[2]
Female	10 (50.0%)	5 (33.3%)	
Male	10 (50.0%)	10 (66.7%)	
**Race:**, n (%)			0.01[2]
White	18 (90.0%)	8 (57.1%)	
Black or African American	0 (0.0%)	4 (28.6%)	
Asian	0 (0.0%)	1 (7.1%)	
Other	2 (10.0%)	1 (7.1%)	
Missing	0	1	
**Ethnicity:**, n (%)			>0.99[2]
Hispanic or Latino	1 (5.0%)	1 (6.7%)	
Not Hispanic or Latino	19 (95.0%)	14 (93.3%)	
**1. How would you describe your current health?**, n (%)			0.41[2]
Excellent	1 (5.0%)	2 (13.3%)	
Good	7 (35.0%)	5 (33.3%)	
Fair	5 (25.0%)	6 (40.0%)	
Very good	3 (15.0%)	2 (13.3%)	
Poor	4 (20.0%)	0 (0.0%)	
**How long have you received dialysis (years)?**			0.42[1]
N	20	13	
Median (IQR)	3.5 (1.1, 5.8)	2.0 (1.0, 5.0)	
**Was the decision to begin dialysis:**, n (%)			>0.99[2]
Planned	9 (45.0%)	7 (50.0%)	
Unplanned	11 (55.0%)	7 (50.0%)	
Missing	0	1	
**4. Did you begin dialysis:**, n (%)			0.74[2]
Inpatient / hospital setting	8 (40.0%)	7 (46.7%)	
Outpatient / clinic setting	12 (60.0%)	8 (53.3%)	
**Are you on a kidney transplant list?**, n (%)			>0.99[2]
No	14 (70.0%)	9 (64.3%)	
Yes	6 (30.0%)	5 (35.7%)	
Choose not to answer	0	1	
**6. Which of the following options were presented to you as treatment options to manage your renal failure? (Check all that apply.) (choice=Peritoneal dialysis)**, n (%)			>0.99[2]

	**DRS Quartiles**		
	**Quartile 1: 0 (N=20)**	**Quartile 4: >25 (N=15)**	**P-value**
Unchecked	7 (35.0%)	5 (33.3%)	
Checked	13 (65.0%)	10 (66.7%)	
**6. Which of the following options were presented to you as treatment options to manage your renal failure? (Check all that apply.) (choice=Home hemodialysis)**, n (%)			<0.001[2]
Unchecked	4 (20.0%)	12 (80.0%)	
Checked	16 (80.0%)	3 (20.0%)	
**6. Which of the following options were presented to you as treatment options to manage your renal failure? (Check all that apply.) (choice=Supportive care without dialysis)**, n (%)			0.20[2]
Unchecked	14 (70.0%)	14 (93.3%)	
Checked	6 (30.0%)	1 (6.7%)	
**6. Which of the following options were presented to you as treatment options to manage your renal failure? (Check all that apply.) (choice=Renal transplant)**, n (%)			0.50[2]
Unchecked	9 (45.0%)	9 (60.0%)	
Checked	11 (55.0%)	6 (40.0%)	
**6. Which of the following options were presented to you as treatment options to manage your renal failure? (Check all that apply.) (choice=None of the above)**, n (%)			1.00[2]
Unchecked	18 (90.0%)	14 (93.3%)	
Checked	2 (10.0%)	1 (6.7%)	
**6. Which of the following options were presented to you as treatment options to manage your renal failure? (Check all that apply.) (choice=Choose not to answer)**, n (%)			
Unchecked	20 (100.0%)	15 (100.0%)	
**7. Who mostly influenced your decision to start dialysis? (choice=Primary care provider)**, n (%)			0.63[2]
Unchecked	18 (90.0%)	12 (80.0%)	
Checked	2 (10.0%)	3 (20.0%)	
**7. Who mostly influenced your decision to start dialysis? (choice=Nephrology provider)**, n (%)			>0.99[2]
Unchecked	8 (40.0%)	6 (40.0%)	
Checked	12 (60.0%)	9 (60.0%)	

	**DRS Quartiles**		
	**Quartile 1: 0 (N=20)**	**Quartile 4: >25 (N=15)**	**P-value**
**7. Who mostly influenced your decision to start dialysis? (choice=Family)**, n (%)			0.14[2]
Unchecked	19 (95.0%)	11 (73.3%)	
Checked	1 (5.0%)	4 (26.7%)	
**7. Who mostly influenced your decision to start dialysis? (choice=Myself)**, n (%)			0.68[2]
Unchecked	16 (80.0%)	13 (86.7%)	
Checked	4 (20.0%)	2 (13.3%)	
**7. Who mostly influenced your decision to start dialysis? (choice=Other)**, n (%)			>0.99[2]
Unchecked	18 (90.0%)	13 (86.7%)	
Checked	2 (10.0%)	2 (13.3%)	
**7. Who mostly influenced your decision to start dialysis? (choice=Choose not to answer)**, n (%)			
Unchecked	20 (100.0%)	15 (100.0%)	
**7. (cont.) If Other, please specify:**, n (%)			>0.99[2]
Death	1 (50.0%)	0 (0.0%)	
Wife	0 (0.0%)	1 (50.0%)	
friends	0 (0.0%)	1 (50.0%)	
life or death waiting for kidney	1 (50.0%)	0 (0.0%)	
Missing	18	13	
**8. How do you currently feel about starting dialysis?**, n (%)			0.50[2]
Best decision I have made	4 (20.0%)	1 (7.7%)	
Not as bad as I thought it would be	8 (40.0%)	4 (30.8%)	
I thought it would be better, but I am okay with it	7 (35.0%)	5 (38.5%)	
I wish I had never started dialysis	1 (5.0%)	1 (7.7%)	
Other	0 (0.0%)	2 (15.4%)	
Missing	0	2	
**8. (cont.) If Other, please specify:**, n (%)			
I'm alive.....	0 (%)	1 (50.0%)	
It's okay, I will live with it	0 (%)	1 (50.0%)	
Missing	20	13	
**9. I feel well prepared for what to expect with dialysis.**, n (%)			0.03[2]
Strongly agree	5 (25.0%)	3 (23.1%)	
Agree	12 (60.0%)	4 (30.8%)	
Undecided	1 (5.0%)	6 (46.2%)	
Strongly disagree	2 (10.0%)	0 (0.0%)	
Missing	0	2	

	DRS Quartiles		
	Quartile 1: 0 (N=20)	Quartile 4: >25 (N=15)	P-value
**10. How did your loved ones feel about your decision?**, n (%)			0.03[2]
Highly satisfied	10 (50.0%)	1 (7.7%)	
Satisfied	9 (45.0%)	9 (69.2%)	
Very unhappy	0 (0.0%)	1 (7.7%)	
Uncertain	1 (5.0%)	2 (15.4%)	
Missing	0	2	
**11. The cost of dialysis played a role in my decision to start dialysis.**, n (%)			0.04[2]
Strongly agree	1 (5.0%)	1 (8.3%)	
Agree	3 (15.0%)	3 (25.0%)	
Undecided	1 (5.0%)	4 (33.3%)	
Disagree	4 (20.0%)	3 (25.0%)	
Strongly disagree	11 (55.0%)	1 (8.3%)	
Missing	0	3	
**14. Has anyone on your care team specifically discussed your prognosis (life expectancy) with you?**, n (%)			0.47[2]
No	10 (50.0%)	8 (66.7%)	
Yes	10 (50.0%)	4 (33.3%)	
Missing	0	3	
**15. How well informed do you feel about you current prognosis (life expectancy)?**, n (%)			0.23[2]
Very well informed	9 (45.0%)	2 (14.3%)	
Somewhat informed	6 (30.0%)	8 (57.1%)	
Unsure	4 (20.0%)	3 (21.4%)	
Somewhat uninformed	1 (5.0%)	1 (7.1%)	
Missing	0	1	
**16. How do you expect your health to be in 12 months from now?**, n (%)			0.57[2]
Much better	4 (20.0%)	3 (23.1%)	
Somewhat better	3 (15.0%)	4 (30.8%)	
The same	10 (50.0%)	6 (46.2%)	
Worse	3 (15.0%)	0 (0.0%)	
Missing	0	2	
**17. If you were seriously ill, would you prefer care to:**, n (%)			>0.99[2]
Extend life, even if it meant more pain and discomfort	11 (61.1%)	8 (66.7%)	
Relieve pain and discomfort, even if it meant not living as long	7 (38.9%)	4 (33.3%)	
Missing	2	3	
**20. It was the right decision**, n (%)			<0.001[2]
Strongly Agree	20 (100.0%)	2 (13.3%)	

	**DRS Quartiles**		
	**Quartile 1: 0 (N=20)**	**Quartile 4: >25 (N=15)**	**P-value**
Agree	0 (0.0%)	7 (46.7%)	
Neither Agree nor Disagree	0 (0.0%)	5 (33.3%)	
Disagree	0 (0.0%)	1 (6.7%)	
**21. I regret the choice that was made**, n (%)			<0.001[2]
Strongly Agree	0 (0.0%)	1 (6.7%)	
Agree	0 (0.0%)	3 (20.0%)	
Neither Agree nor Disagree	0 (0.0%)	8 (53.3%)	
Disagree	0 (0.0%)	3 (20.0%)	
Strongly Disagree	20 (100.0%)	0 (0.0%)	
**22. I would go for the same choice if I had to do it over again**, n (%)			<0.001[2]
Strongly Agree	20 (100.0%)	3 (20.0%)	
Agree	0 (0.0%)	6 (40.0%)	
Neither Agree nor Disagree	0 (0.0%)	5 (33.3%)	
Disagree	0 (0.0%)	1 (6.7%)	
**23. The choice did me a lot of harm**, n (%)			<0.001[2]
Strongly Agree	0 (0.0%)	3 (20.0%)	
Neither Agree nor Disagree	0 (0.0%)	5 (33.3%)	
Disagree	0 (0.0%)	7 (46.7%)	
Strongly Disagree	20 (100.0%)	0 (0.0%)	
**24. The decision was a wise one**, n (%)			<0.001[2]
Strongly Agree	20 (100.0%)	2 (13.3%)	
Agree	0 (0.0%)	7 (46.7%)	
Neither Agree nor Disagree	0 (0.0%)	6 (40.0%)	

[1]Kruskal-Wallis p-value; [2]Fisher Exact p-value;
